# Ins and outs of endovenous laser ablation: afterthoughts

**DOI:** 10.1007/s10103-013-1499-7

**Published:** 2014-01-08

**Authors:** H. A. Martino Neumann, Martin J. C. van Gemert

**Affiliations:** 1 Department of Dermatology, Erasmus University Medical Center, Rotterdam, The Netherlands; 2 Department of Biomedical Engineering and Physics, Academic Medical Center, University of Amsterdam, Amsterdam, The Netherlands

**Keywords:** Endovenous laser ablation, Hemoglobin and water absorption targeting, LEED

## Abstract

Physicists and medical doctors “speak” different languages. Endovenous laser ablation (EVLA) is a good example in which technology is essential to guide the doctor to the final result: optimal treatment. However, for the doctor, it is by far insufficient just to turn on the knobs of the laser. He should understand what is going on in the varicose vein. On the other hand, the physicist is usually not aware what problems the doctor finds on his road towards improving a new technique. We have tried to bring both languages together in the special on *Ins and outs of endovenous laser ablation* published in this issue of Lasers in Medical Science. The 13 articles include endovenous related clinical (de Roos 2014; Kockaert and Nijsten 2014; van den Bos and Proebstle 2014) and socioeconomical articles (Kelleher et al 2014), the first paper on the molecular pathophysiologic mechanisms (Heger et al 2014), fiber tips (Stokbroekx et al 2014), the future of EVLA (Rabe 2014), a review of EVLA with some important issues for debate (Malskat et al 2014), an excellent paper on transcutaneous laser therapies of spider and small varicose veins (Meesters et al 2014), as well as several scientific modeling articles, varying from a mathematical model of EVLA that includes the carbonized blood layer on the fiber tip (van Ruijven et al 2014) and its application to the simulation of clinical conditions (Poluektova et al 2014) via experimental measurements of temperature profiles in response to EVLA, radiofrequency waves, and steam injections (Malskat et al 2014) to a literature review and novel physics approach of the absorption and particularly scattering properties of whole blood also including the infrared wavelengths used by EVLA (Bosschaart et al 2014). The aim of our afterthoughts, the 14th article in this special, is to try to amalgamate the clinical and physical contents of these contributions, providing the reader with the bridge that overlaps these different backgrounds.

## Introduction

Lasers are used in medicine since the late 1960s for the treatment of port wine stains, so it was an excellent but also logical idea to bring laser light into blood vessels. Seldinger already demonstrated years before the access to blood vessels for selective angiography [14]. The capillaries of vascular skin malformations are too thin to work out this concept of intracapillary laser treatment. However, arteries as well as varicose veins, the latter with diameters of 3 to over 20 mm for the incompetent greater saphenous vein (GSV), are easy to access through a puncture in the skin and wide enough to introduce a fiber catheter. In the 1980s, laser angioplasty developed aimed to open up stenosed or obliterated peripheral and coronary arteries [15, 16]. Subsequently, in the late 1990s, the phlebologic community was ready to apply laser light in varicose veins, albeit now aimed to permanently *obliterate* the vein, stimulated by a huge amount of varicose vein patients who were waiting for a good alternative to the nearly 100 years old stripping operation [17]. In contrast to the development of drugs, where very strict regulations exist for a full development trajectory, in surgery this is not required for technological innovations. So, the first laser treatment in a varicose vein was performed in Spain in January 1998 [18]. The laser catheter and fiber, and even a small endoscope, were introduced in the GSV; the laser was turned on and the fiber slowly pulled back with excellent results. The first laser was chosen because of its easy access, a diode laser with a wavelength of 810 nm which is absorbed by the hemoglobin content of the blood. It quickly turned out that intravascular coagulation of the varicose vein by laser light was patient friendly mainly due to the absence of general anesthesia and highly effective. As it is a daycare procedure, laser ablation was significantly cheaper than the classical stripping operation. Another technique developed in dermatology by Jeffrey Klein for liposuction is the tumescent anesthesia [19]. The introduction of Klein's solution into the saphenous compartment provides anesthesia as well as compression by the tumescent fluid, thus, also a reduction of the vein diameter.

So, at the start of this millennium, several ideas and technical possibilities came together and created the venous endovascular laser procedure. Looking back, it is still amazing to observe the rapid evolution of this technique, now known as endovascular laser ablation (EVLA), even though it was without much, if any, knowledge of the processes which are going on during and after the treatment.

Just like in the nineteenth century when every self-respecting gynecologist had developed its own forceps for complicated deliveries, phlebologists started to change the laser probe, wavelength, laser power, and pullback velocity.

At the time that insurance companies already began to refund EVLA procedures to their clients, some phlebologists woke up. Among them was Thomas Proebstle, a German dermatologist with a previous Masters in Physics [20, 21]. Many theories were subsequently launched to explain the effectiveness of EVLA. The next step came also from a clinician. Renate van den Bos, dermatologist in Rotterdam, performed a meta-analysis in which she identified that the available clinical data showed EVLA to be superior to surgery and foam sclerotherapy [22]. In her group, studies were started to explain the effects of heat conduction and to investigate the consequences of coagulation of blood and subsequent carbonization around the laser tip [23]. The Duplex ultrasound observation of bubbles in front of the laser tip turned out to be steam bubbles which play a major role in the (convective) transfer of heat to the luminal surroundings, including the vein wall, suggested to behave comparable to the industrial model of heat transport in heat pipes [20, 24, 25]. All these observations suggested that heat development and heat transport from the source (laser light emitted out of the fiber) to the sink (vessel wall) are an essential part of the clinical success of EVLA. Nevertheless, the exact mechanisms of heat transport which take place at the laser fiber tip and propagate through the blood to the vessel wall still remained incompletely understood, which stimulated teams of clinicians and physicists to join forces in combined projects trying to unravel the secrets behind EVLA. Unfortunately, the number of such teams active in EVLA remained limited [24–27].

It is not surprising therefore that the level of phlebology-related physics was seriously criticized in the review of EVLA in this issue [8], and it was hypothesized that lack of awareness of this problem stimulated the unsubstantiated promotion of new laser wavelengths for EVLA procedures. The two examples used to demonstrate this convincement were the suggested superiority of water over hemoglobin as EVLA absorption targets, and the suggested importance and widespread use of Joules per centimeter vein length, termed linear endovenous energy density (LEED), aimed to characterize the technical parameters of EVLA therapy so the procedure used can be exactly duplicated if needed.

Our afterthoughts will cover the five currently proposed mechanisms of EVLA efficacy, the irrelevance of water versus hemoglobin absorption targeting, the uselessness of reporting Joules per centimeter (LEED), the value of computational modeling, and the timing of randomized controlled trials (RCTs).

## The five proposed mechanisms of action for EVLA

Five mechanisms have now been identified that at least theoretically contribute to the efficacy of EVLA. The fifth is a novel identified mechanism in this issue [5]. The first four are the long-term consented thermal laser–tissue interaction mechanisms that cause the temperature of the vein wall to increase, the assumed thermal key mechanism of EVLA efficacy. First, direct contact between fiber tip and vein wall [28]. Second, thermal interactions between the laser light emitted out of the fiber tip and the vein wall. This mechanism has two components: (a) direct absorption by the vein wall of the light scattered by the blood that reaches the wall, leading to an increased vein wall temperature and (b) heating up the blood surrounding the fiber tip by direct laser light absorption which causes heat flows to conduct towards the wall and, upon arrival, produce an increased wall temperature. In phlebology, only mechanism (a) was considered; however, we showed in this issue that (b) actually dominates over (a) [11]. Neglecting part (b) directed the whole EVLA field into the presumed but unproven greatness of the water absorption wavelengths. Third, the effects of steam bubbles, which were touted but not proven to be the main EVLA mechanism [20, 24], including the additional suggestion that the vein acts comparable to an industrial heat pipe [25], and fourth, the effects of the carbonized blood layer glued on the fiber tip. This mechanism was widely known for many years to result from switching on the laser while its fiber was embedded in the blood [29]. Because the black layer strongly absorbs the emitted light, it becomes exceedingly hot, around 1,000 °C, and causes damage or even melting of the tip [24]. The consequential heat flow from the hot tip towards the vein wall is capable of causing irreversible wall injury [23, 30]. Submission and publication of this long-known [29] but for the EVLA clinical community apparently novel mechanism provoked very strong phlebologic opposition in part because a black layer absorbs all wavelengths equally well, thus suggesting virtually no influence of wavelength on EVLA efficacy, which challenged the general accepted belief at that time that water absorption based wavelengths were the way to go, and consequently, therefore, also jeopardized possible commercial interests.

**Fig. 1:**
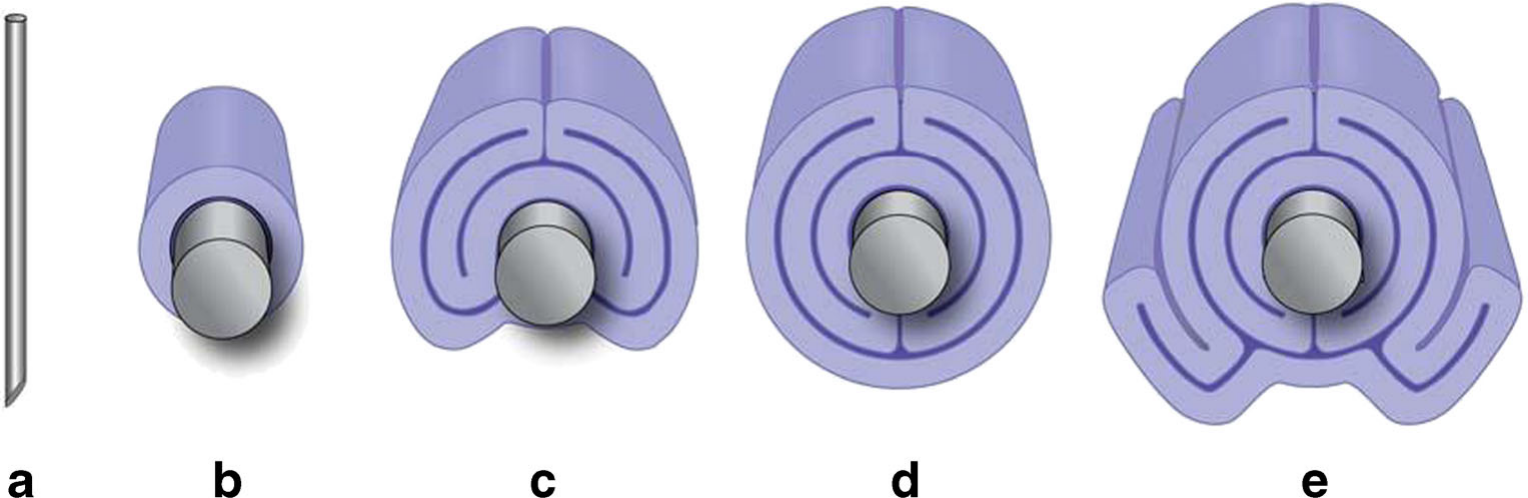
Possible folding geometries of the (GSV) vein wall around the EVLA fiber catheter following tumescent fluid injection for several vein diameters. The catheter, shown as the inside rod, has an assumed diameter of 3 mm, the (*blue displayed*) vein wall an assumed thickness of 1 mm. The possible reduction in wall thickness due to the increased pressure by the tumescent fluid has been neglected. We define FWDR as the ratio of fiber diameter and inner vein wall circumference (π times the inner vein diameter). From left to right: **a** fiber with 0.6 mm diameter, **b** vein with an inner wall diameter of about 3 mm and FWDR = 6.4 %; **c** inner wall diameter of about 10 mm and FWDR = 1.9 %; **d** inner wall diameter of about 15 mm and FWDR = 1.3 %; **e** inner wall diameter of about 20 mm and FWDR = 0.95 %. For **c** and **e**, other folding arrangements are possible (not shown). Inner vein wall diameters between 3 and 15 mm will always include a combination of 1 and 3 wall thickness layers around the 3 mm diameter catheter. Inner vein wall diameters between 15 and 27 mm always include 3 and 5 wall thickness layers around the catheter. An inner vein diameter of about 27 mm has five layers around the whole catheter surface (not shown). Illustrations by Ron Slagter, Haarlem, The Netherlands

The fifth is a novel mechanism for the first time identified in Heger's paper in this issue [5]. This fifth mechanism is not based on thermal injury of the vein *wall* but on the sequelae of thermal injury of *blood*, forming coagula within the vein lumen. Despite doubts expressed in Heger's paper on the likelihood of having sufficient blood volume in an EVLA-treated vein to make this mechanism an important contributor to EVLA, we nevertheless include this novel mechanism here in view of the fact that it contributes at least theoretically to EVLA efficacy. This is in the spirit of two other proposed mechanisms that may unlikely dominate the efficacy of EVLA despite suggested importance, i.e., direct laser light absorption by the vein wall [26] and direct contact between fiber tip and vein wall [28]. Concerning the former, comparing zero with normal vein wall absorption in our model simulations gave similar computed wall temperatures, implying a limited importance of direct vein wall light absorption [11]. Concerning the latter, the small vein wall injury line of about 0.6 mm width relative to the vein wall circumference of about 10 to 60 mm (diameters between 3 and 20 mm) was suggested inadequate for vein obliteration [8]. Figure 1 visually supports this idea. So far, it is unknown to us whether, and if so how, this fifth mechanism may provide opportunities for an improved EVLA procedure or novel fiber tip design.

Interestingly, this wealth of five action mechanisms perhaps implies that overtreatment occurs which, if true, may explain the immense efficacy of any EVLA procedure currently used, seemingly independent of anything, i.e., wavelength, power, pullback velocity, and vein diameter. More importantly, perhaps, it may also imply that measures exist that retain the efficacy but reduce the pain experienced during and/or following the procedure. An interesting recent observation is that EVLA causes *postoperative* pain whereas endovenous steam ablation causes pain *during* treatment [31]. Because both therapies use the same tumescent anesthesia, this observation has currently no explanation. Thus, the issue of pain reduction management at equal EVLA efficacy is not well understood but is in our opinion a fascinating route to pursue in the future.

## The irrelevance of water versus hemoglobin absorption targeting

Insufficient knowledge of the fact that laser–tissue interaction mechanism (b), vein wall injury by heat flows originating from the direct heated blood, also occurs in addition to heating of the vein wall by direct light absorption, turned EVLA into a turmoil of new EVLA wavelengths that use the water content of the vein wall as the absorption target even to the extent of touting 1,950 nm, indeed very strongly absorbed by water, as a wavelength where EVLA may perhaps not need tumescent anesthesia [32]. These predictions evidently neglected the significant absorption by the water content of blood, well-known to be about 60 %, for the photons traveling from the fiber tip to the vein wall. It is perhaps interesting to speculate what would have happened if such knowledge would have been realized earlier. We hypothesize that this could have prevented the repeated introduction of new wavelengths. However, we also submit that this could have equally prevented gaining the important knowledge of the effects of the various wavelengths that is available to date, particularly their small, if any, differences in EVLA efficacy.

## The uselessness of linear endovenous energy density (LEED)

LEED (Joules per centimeter vein length) was initiated in 2005 [33]. Malskat et al. [8] explain that the unit Joules per centimeter originates from the ratio of laser power and pullback velocity, two essential elements to characterize the procedure and that, by taking this ratio, insufficient information remains that allows exactly repeating the procedure.

An interesting question now is how it became possible that phlebology-interested clinicians, as well as some physicists, embraced a parameter that was aimed to characterize the procedure but fails to do so without ever noticing. Our most likely explanation is that clinicians, unlike physicists, have not been thoroughly “programmed” in feeling the difference between laser *energy* and laser energy given per second, the laser *power*. In Malkat's paper, an attempt is made to tutor the reader on this difference [8].

The thermal efficacy of EVLA is clearly driven by the laser *power*, not the laser energy [8, 10, 34], because the laser action is virtually continuous (for laser pulses shorter than about 0.1 s per pulse, it would have been the laser energy per pulse). A clarifying example, perhaps somewhat ludicrous, is to consider a 50 Joules/cm EVLA procedure. This LEED produces an effective EVLA outcome if given as a 10 Watt laser power and a 2 mm/sec pullback velocity, but it can equally be given as 0.1 Watt and 0.02 mm/sec, here without much hope of an effective EVLA procedure. Even 50 Joules/cm can be given by extreme circumstances for EVLA procedures, e.g., as 10,000 Watt requiring 200 cm/sec pullback velocity (0.15 sec pullback for a 30-cm vein length) and likely effective, or without any effect as 0.001 Watt and 0.0002 mm/sec (417 hours pullback for a 30 cm vein length!). The latter examples obviously are clinically not very feasible, but we hope they demonstrate the uselessness of using Joules per centimeter because both still deliver the 50 Joules per cm vein length!

Thus, using Joules per centimeter makes no sense, neither clinically nor scientifically, and the cleverly chosen fancy name of linear endovenous energy density may have promoted this “unit of phlebology” into its dubious status of importance. The other interesting question now is whether the clinical use of LEED did hurt the field of EVLA? Probably, it did and still does. When power and pullback velocity are given in a paper, providing a number for LEED is useless but at least the procedure can exactly be repeated by other clinicians if wanted. However, it has also triggered a lack of feeling the necessity of giving these essential parameters which then precludes the possibility of repetition by others.

## The value of computational modeling

The computational models currently available for EVLA [10, 34] assume the laser fiber to be exactly positioned in the middle of a perfectly cylindrical venous tube, and emission of the laser light power out of the fiber tip according to a point source, which redistributes itself radially by a diffusion process, governed by the blood's scattering and absorption properties at the wavelength considered. Our model also includes the carbonized layer of blood [10] which absorbs about 45 % of the laser power [30], the source for the very high computed temperature of around 1,000 °C for this layer. The laser light power per very small area, called the fluence rate (Watt per area), available in the vein lumen, the vein wall and perivenous tissue, can be absorbed according to their respective absorption coefficients, and the temporospatial temperature distribution follows from the solution of the bioheat equation, e.g., see our review [8]. Although the models are idealized, the majority of mechanistic concepts of EVLA are correctly incorporated, with the exception of the thermal effects of steam bubbles and the wound healing effects of the blood coagula on the vein wall. The value of modeling then is its prediction of clinical behavior for varying EVLA parameters. Such parameter variations, e.g., power, pullback velocity, vein diameter, and tissue absorption (e.g., water versus hemoglobin targeting wavelengths) cannot very easily be accounted for during clinical or experimental procedures. However, although the results can only be considered as simulations of an idealized reality, the irrelevance of water versus hemoglobin absorption targeting, the negligible contribution of direct vein wall light absorption, the effects of a reduced vein diameter, and the uselessness of Joules per centimeter follow perfectly from these models.

## The timing of RCTs: now or later?

RCTs are not only the basis of modern evidence-based medicine but also for providing guidelines and reimbursement by insurance companies. There are RCTs available in literature in which EVLA is compared with radiofrequency thermal ablation, foam sclerotherapy, classical surgery, and recently endovenous steam ablation. As laser parameters are widely different, a comparison let alone a meta-analysis is virtually impossible. Standardization of the EVLA technique thus is essential for further RCTs.

One RCT that requires carrying out *now* is comparing the EVLA efficacy of two (or more) laser wavelengths while keeping all other parameters exactly the same. This study is underway in Rotterdam, and the results are expected to become available mid-2014. The issue of designing additional RCTs likely is hampered by our lack of knowledge of the true effects of steam bubbles. Here, we propose that the answer has to await full incorporation of steam bubbles in future computational modeling, so predictions of their effects can be made which may then stimulate the design of relevant RCTs to test the predicted outcomes.

Chronic venous diseases and particularly varicose veins are a socioeconomic burden [4]. Society could therefore expect that with the development of new therapies for such a common problem, achieving an excellent cost-benefit ratio is mandatory. Finally, the patients themselves has great influence. So, patient-related outcome parameters should support the value of new treatments. In this respect, although EVLA is very effective in the obliteration of incompetent veins, foam sclerotherapy, endovenous radiofrequency ablation, and endovenous steam ablation turn out to be better in patient-related outcome parameters. As we mentioned before, it is plausible that with the current laser settings used in EVLA procedures, we overdose our target leading to vessel injuries, extravasation of blood, and postoperative pain. The fact that EVLA is so effective in the primary outcome parameter (occlusion of the incompetent vein) may have prevented carrying out dose finding studies, which are mandatory in pharmacotherapy. It is our belief that the side effects of EVLA can be strongly reduced, and by this, an improvement of patient-related outcome parameters can be achieved by a good dose finding study concerning the laser settings.

## Finally

The cooperation between physics and phlebology has opened doors that one alone never could even have unlocked. Although EVLA is just 16 years old [18, 28, 35, 36], we are still not fully aware of all that happens during a procedure, but we now at least have the duty to explain what currently is known of this elegant and effective technique to those who perform this treatment on a regular basis in their practice. To demystify the superior influence of the chosen laser parameters, everybody who performs EVLT should realize that the introduction of laser light (*power* rather than energy) into the blood (mainly water and hemoglobin as absorbing chromphores) will result in a reaction in which heat is essential, but it is just this rise in temperature which makes the procedure difficult to understand completely because clotting of blood and carbonization on the fiber tip take place. A heat-induced blood coagulum will be formed that behaves differently than a “normal” thrombus. Nevertheless, it will release many active substances which will interact with the vessel wall. Even in case this vessel wall is not yet affected by heat, the active lytic enzymes and other elements from the blood coagulum will influence the vessel wall [5]. A spontaneous thrombosis in the GSV usually recanalizes spontaneously too, leaving in the end a patent vein. However, in EVLA, obliteration is the end stage achieved by the combined action of thrombus formation and heat conduction. Coagulum and/or thrombus formation during treatment for varicose veins is not only unique for EVLA but also for other thermal ablations such as by radiofrequency waves and steam. Also, foam sclerotherapy forms a kind of coagulum and sometimes even thrombus formation occurs. Nevertheless, in EVLA, it is an essential part of the process because a pure bloodless vessel is a utopia and intravenous laser light is very effective in forming coagula, irrespective of wavelength. In addition, when the laser is switched on a coagulum will be formed around the fiber tip and will be partly transformed into carbon by the extreme high temperature reached at the laser tip (>1,000 °C). Carbon is black and consequently absorbs all EVLA laser wavelengths equally well. Certainly, not all laser power will be absorbed by the carbon layer, but from this moment on, EVLA has reduced its possible wavelength dependent efficacy and strongly increased the intraluminal production of heat.

In conclusion, the prognosis of an increasing incidence of age-related varicose veins [7] stimulates us to extend our knowledge of what is really going on during and after EVLA, so there is a need for new research, clinical as well as in the laboratory. Only then will we be able to come up with a consented best therapy that includes not only a very high success rate but also a strong reduction or even absence of pain during as well as after EVLA. If the already proven fruitful cooperation between doctor and physicist continues, this already good therapy can reach the status of excellence.
